# Effects of transcutaneous auricular vagus nerve stimulation on cardiorespiratory fitness in patients with subacute stroke: a study protocol for a randomized controlled trial

**DOI:** 10.3389/fcvm.2026.1835286

**Published:** 2026-08-11

**Authors:** Xinyuan Han, Zhijun Huang, Yu Li, Na Zhang, Ziting Gao, Jing Ning, Yangyang Feng

**Affiliations:** Neurological Rehabilitation Department, Shaanxi Provincial Rehabilitation Hospital, Xi'an, China

**Keywords:** cardiorespiratory fitness, peak oxygen uptake, randomized controlled trial, stroke, transcutaneous auricular vagus nerve stimulation

## Abstract

**Background:**

Patients with stroke often experience reduced cardiorespiratory fitness, which increases the risk of cardiovascular events, and conventional rehabilitation has limited efficacy in improving it. Transcutaneous auricular vagus nerve stimulation (taVNS) can modulate autonomic function, but its effect on cardiorespiratory fitness in stroke patients remains unclear.

**Methods:**

This randomized controlled trial will enroll 160 patients with subacute stroke, who will be randomly assigned in a 1:1 ratio to an experimental group or a control group. Both groups will receive conventional rehabilitation training. The experimental group will additionally receive active taVNS (left auricular concha, 25 Hz, 200 µs, intensity set at or slightly above the sensory threshold, 30 min/session, 5 sessions/week for 4 weeks), while the control group will receive sham stimulation (0 mA). The primary outcome is set as peak oxygen uptake (VO_2_peak), assessed at baseline (T0), post-intervention (T1), and follow-up (T2). Secondary outcomes include: (1) inflammatory markers (IL-6, TNF-α); (2) neurological function (National Institutes of Health Stroke Scale, NIHSS); and (3) activities of daily living (modified Barthel Index, MBI), all measured at T0, T1, and T2. Resting-state electrocardiogram (5 min) will be recorded at T0, T1, and T2 to analyze heart rate variability (HRV) as an exploratory outcome to elucidate autonomic mechanisms, without multiplicity correction. Data will be analyzed following the intention-to-treat principle.

**Discussion:**

This protocol aims to investigate the effect of taVNS on cardiorespiratory fitness in patients with subacute stroke.

## Introduction

Stroke is a leading cause of disability and mortality among adults in China, imposing a substantial burden on society and families due to its high incidence. Patients in the subacute phase often face multiple functional impairments, among which the decline in cardiorespiratory fitness has received insufficient attention (1). This decline stems not only from stroke-induced damage to central autonomic regulation (2) and cardiac function (3), but also from secondary prolonged bed rest and physical inactivity (4, 5). Cardiorespiratory fitness, with peak oxygen uptake (VO_2_peak) as its core indicator, is a powerful predictor of overall functional reserve (6). For stroke survivors, low cardiorespiratory fitness not only hinders the recovery of motor function and activities of daily living but is also associated with adverse cardiovascular prognosis (7, 8). Therefore, identifying effective rehabilitation interventions to improve cardiorespiratory fitness in patients with subacute stroke has become a noteworthy issue in this field.

High-intensity exercise is an effective means of improving cardiorespiratory fitness (9). However, common functional impairments following a stroke often prevent patients from achieving the required exercise intensity. In recent years, transcutaneous auricular vagus nerve stimulation (taVNS), a non-invasive neuromodulation technique, has gained widespread attention due to its safety and efficacy. Its theoretical basis lies in the superficial distribution of the auricular branch of the vagus nerve in the auricular concha. Low-intensity electrical stimulation in this area can antidromically activate the vagus nerve, modulate key brainstem nuclei such as the nucleus tractus solitarius, and optimize autonomic nervous system function (10, 11). Preliminary studies have suggested the potential of taVNS in improving post-stroke cognitive and motor functions, as well as in treating conditions such as epilepsy and depression (12–17). Mechanistically, taVNS may, by modulating autonomic function, improve cardiac chronotropic function and reduce inflammatory responses, thereby enhancing cardiorespiratory fitness (18, 19). However, no high-quality randomized controlled trial to date has specifically investigated the effect of taVNS on cardiorespiratory fitness in stroke patients.

To accurately assess cardiorespiratory fitness, this study adopts the internationally recognized cardiopulmonary exercise test (CPET) (20). CPET provides a comprehensive evaluation of the integrated function of the heart, lungs, and skeletal muscles by simultaneously monitoring gas exchange under progressively increasing exercise loads, with VO_2_peak as its core measure (21). Under close supervision, CPET can be safely performed in patients with subacute stroke to precisely quantify the degree of cardiorespiratory impairment and the efficacy of interventions.

This study aims to systematically evaluate, through a methodologically rigorous randomized controlled trial, the effects of a 4-week taVNS intervention combined with conventional rehabilitation training on cardiorespiratory fitness in patients with subacute stroke. We hypothesize that, compared with the sham stimulation control group, active taVNS will significantly improve VO_2_peak, along with favorable changes in heart rate variability, inflammatory markers, and neurological function.

Notably, cardiovascular events are a leading cause of death among stroke survivors (22). The field of neurocardiology emphasizes the bidirectional interactions between the brain and the heart. Cardiorespiratory fitness, as a powerful predictor of cardiovascular outcomes (6), may serve as an integrative marker linking neurological recovery and cardiac prognosis after stroke.

## Methods

### Study design

**Figure 1:**
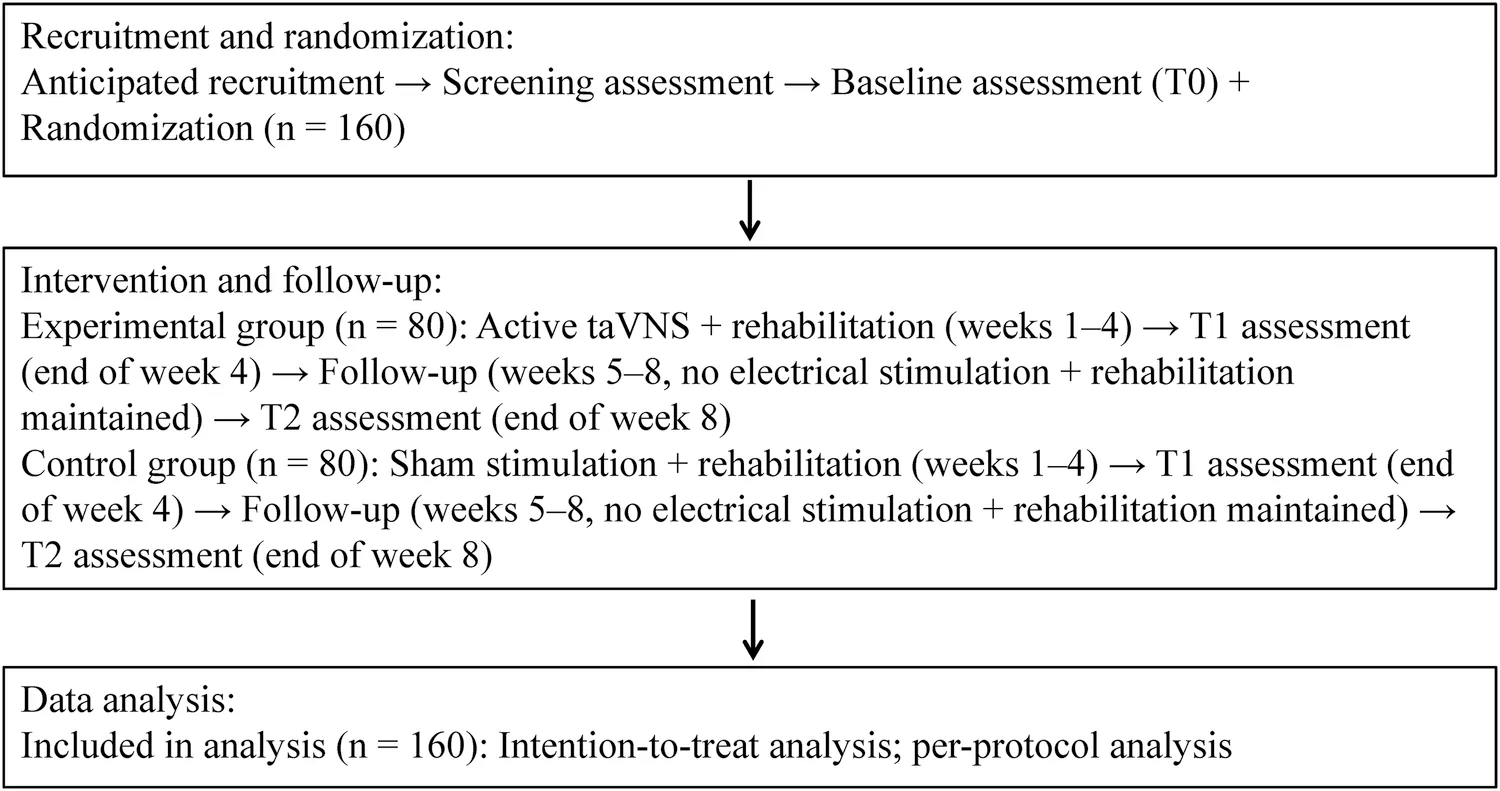
Anticipated participant flow diagram (CONSORT framework). T0 = baseline assessment point; T1 = post-intervention assessment point (end of week 4); T2 = follow-up assessment point (end of week 8). During the follow-up period (weeks 5–8), no electrical stimulation is delivered; only conventional rehabilitation is maintained. The final report will include the actual numbers of participants screened, excluded, randomized, lost to follow-up, and included in each analysis.

This study is a randomized, sham-controlled, parallel-group superiority trial. The study procedures and schedule are detailed in Table 1 (Study schedule and assessment timeline) and Figure 1 (Anticipated participant flow diagram).

**Table 1:** Study schedule and assessment timeline.

Item	Assessment point T0 (Baseline)	Intervention phase (weeks 1–4)	Assessment point T1 (end of intervention)	Follow-up phase (weeks 5–8)	Assessment point T2 (follow-up assessment)
Time correspondence	Week 0	← 4 weeks →	End of Week 4	← 4 weeks →	End of Week 8
Enrollment and allocation					
Participant screening	●				
Informed consent	●				
Demographic data collection	●				
Medical history and medication record	●				
Randomization	●				
Intervention					
Experimental group (active taVNS)		●			
Control group (sham stimulation)		●			
Conventional rehabilitation training		●		●	
Primary outcome					
Cardiopulmonary exercise testing (VO_2_peak)	●		●		●
Secondary outcomes					
Inflammatory markers (IL-6, TNF-α)	●		●		●
NIHSS score	●		●		●
Modified Barthel index	●		●		●
Exploratory outcome					
Heart rate variability (SDNN, HF)^1^	●		●		●
Safety assessment					
Adverse event recording		●	●	●	●
Serious adverse event reporting		●			
Compliance monitoring					
Treatment session completion record		●	●		
Others					
Concomitant medication record	●	●	●	●	●
Unblinding request record		●	●	●	●

1 Heart rate variability (HRV), as an exploratory outcome, will be analyzed from 5 min resting-state electrocardiograms recorded before cardiopulmonary exercise testing at T0, T1, and T2. It will not be subjected to multiplicity correction and will be used only for explanatory analyses. T0 = baseline assessment point (pre-intervention assessment and randomization); T1 = post-intervention assessment point (end of week 4); T2 = follow-up assessment point (end of week 8). The intervention phase spans weeks 1–4, and the follow-up phase spans weeks 5–8. The symbol “●” indicates that the procedure or assessment is performed at the corresponding time point.

### Participant recruitment

Participants will be recruited consecutively from July 2026 to June 2027. Recruitment sources will include patients attending the Neurorehabilitation Center of Shaanxi Rehabilitation Hospital and community-dwelling stroke survivors recruited from relevant communities in Xi'an City.

### Diagnostic criteria

The diagnosis of stroke will conform to the World Health Organization (WHO) definition. All cases must be confirmed as acute ischemic or hemorrhagic stroke by cranial computed tomography (CT) or magnetic resonance imaging (MRI). The diagnosis will be independently verified by at least two specialist neurologists.

### Inclusion criteria

(1) Age 40–75 years;
(2) First-ever stroke, disease duration 1–6 months;
(3) Presence of definite lower limb hemiparesis: Brunnstrom stage ≥II in the affected lower limb, with or without upper limb involvement;
(4) Clinically stable condition, stable vital signs, and clear consciousness;
(5) Possess the minimum motor function required to complete cardiopulmonary exercise testing: be able to perform a pedaling motion in a seated position, have a muscle strength of the affected lower limb ≥grade 3, and be capable of safely completing cycle ergometer-based cardiopulmonary exercise testing;
(6) Voluntary provision of written informed consent.

### Exclusion criteria

(1) Presence of severe cardiac dysfunction (LVEF < 40% or NYHA class ≥ III), hepatic dysfunction (ALT/AST > 3 × upper limit of normal), or renal dysfunction (eGFR < 30 mL/min/1.73 m^2^); uncontrolled hypertension (resting systolic blood pressure ≥180 mmHg or diastolic blood pressure ≥110 mmHg); unstable angina, recent (<3 months) myocardial infarction, symptomatic valvular heart disease, atrial fibrillation, high-risk ventricular arrhythmias, second-degree type II or higher atrioventricular block, or symptomatic bradycardia (<45 bpm); acute coronary syndrome; chronic obstructive pulmonary disease; or active infection.
(2) Presence of severe mental illness or cognitive impairment;
(3) Skin lesions, infection, or deformity of the ear;
(4) Affected lower limb muscle strength ≤grade 2, and inability to complete cycle ergometer-based cardiopulmonary exercise testing;
(5) Presence of implanted electronic medical devices (including pacemakers, implantable cardioverter-defibrillators, neurostimulators, deep brain stimulators, and other active implantable electronic devices), as they may be susceptible to electrical interference or cannot be safely used in the taVNS environment;
(6) Current participation in other clinical trials that may interfere with the outcomes of this study.

### Screening procedures

This study plans to recruit 160 participants with subacute stroke from the Neurorehabilitation Center of Shaanxi Rehabilitation Hospital and community-dwelling stroke survivors in Xi'an. All potential participants must have their clinical diagnosis independently confirmed by at least two neurologists according to the diagnostic criteria of the WHO and the American Heart Association/American Stroke Association (AHA/ASA), and confirmed by cranial CT or MRI. Additionally, participants must present with definite lower limb hemiparesis (Brunnstrom stage ≥II in the affected lower limb), with or without upper limb involvement. Individuals passing the initial screening will undergo a systematic baseline evaluation conducted by a trained research coordinator. This evaluation includes assessments of motor function, autonomic symptoms, and safety screening for cardiopulmonary exercise testing. Eligibility will be finalized by checking against the inclusion and exclusion criteria checklist. All potential participants will undergo systematic screening for CPET contraindications before enrollment, including medical history and physical examination. If pulmonary embolism or deep vein thrombosis is suspected, further investigations (e.g., four-limb deep venous ultrasound, pulmonary CTA) will be performed to ensure CPET safety.

### Discontinuation and withdrawal criteria

Participants will discontinue treatment or be withdrawn from the study if any of the following occur:

(1) Occurrence of a serious adverse event that, in the investigator's judgment, makes continued participation inappropriate. Such participants will discontinue treatment, but their available data will still be included in the intention-to-treat analysis;
(2) Participant voluntarily withdraws informed consent and requests withdrawal. Such participants will be withdrawn from the study, and all data collection will cease;
(3) Poor adherence: The actual number of completed intervention sessions is less than 80% of the planned number. Such participants will discontinue treatment but will still be encouraged to continue participating in subsequent follow-up assessments, and their data will be included in the intention-to-treat analysis;
(4) Receipt of other interventions during the study period that may affect the outcome measures. Such participants will discontinue treatment, but their available data will still be included in the intention-to-treat analysis;
(5) Loss to follow-up (missed two consecutive scheduled visits or unable to be contacted). Such participants will not be considered as having actively withdrawn from the study; their available data will be included in the intention-to-treat analysis, and missing data will be handled using multiple imputation.

### Randomization and allocation

This study will use a stratified block randomization method, with stratification factors including age (≤60 years vs. >60 years) and disease duration (≤3 months vs. >3 months). A statistical specialist not involved in clinical procedures or outcome assessments will generate the random sequence using R software. The 160 participants will be allocated in a 1:1 ratio to the taVNS group (*n* = 80) or the sham stimulation group (*n* = 80). To conceal allocation, the random sequence will be placed in sequentially numbered, opaque, sealed envelopes. Researchers will open the envelopes in strict order of participant enrollment and will meticulously record the date, time, and signature of the person opening each envelope.

### Allocation and blinding

This study employs a partial blinding design: participants, responsible clinicians, outcome assessors, and statistical analysts will remain blinded to group allocation, while the intervention providers cannot be blinded due to the visibility of the stimulation site (auricular concha). The intervention providers, rehabilitation therapists, responsible clinicians, and outcome assessors are independent personnel with separate roles; rehabilitation therapists are responsible for conventional rehabilitation training and are not involved in study assessments. An independent research coordinator, who is fully aware of the group allocation, will be responsible for random assignment and allocation concealment. In the event of a serious adverse event, the responsible clinician will decide whether to unblind, and will record the time, reason, and signature of unblinding. The unblinding information will be disclosed only to the extent necessary for treatment. The rehabilitation therapists will remain blinded to group allocation; they will adjust rehabilitation intensity solely based on Brunnstrom stage and modified Barthel Index scores, and will not be involved in the administration of taVNS or sham stimulation, nor have access to group allocation information.

To evaluate the effectiveness of blinding, at the end of each week during weeks 1 to 4 (once per week), a research assistant who is not involved in treatment administration will ask each participant: “Do you think you received active stimulation or sham stimulation in this session?” The response options are active stimulation, sham stimulation, or uncertain. The participant's confidence in their answer will be recorded on a 10-point scale. For analysis, responses of “uncertain” will be excluded, and the proportion of correct guesses will be calculated based only on the two definitive responses (“active stimulation” or “sham stimulation”) as the number of correct guesses divided by the total number of definitive responses. A binomial test will be used to determine whether this proportion is significantly higher than 50% (*α* = 0.05). The proportion of “uncertain” responses will be reported separately as a supplementary indicator of blinding uncertainty. If the correct guess rate at all time points is close to 50%, blinding will be considered successful. If the correct guess rate at any time point is significantly higher than 50%, it will suggest potential blinding failure; this result will be used to quantify the potential impact of expectancy bias (e.g., on CPET performance), considered in the interpretation of the final results, and reported as a limitation. It should be emphasized that this assessment is intended only to monitor blinding effectiveness and cannot substitute for a credible sham stimulation design.

### Intervention

A transcutaneous auricular vagus nerve stimulation device manufactured by Jiangxi Jingyi Medical Technology Co., Ltd., China, will be used. Stimulation electrodes will be placed over the vagus nerve distribution hotspot in the left auricular concha (23, 24). The stimulation parameters are: biphasic square wave, frequency 25 Hz, pulse width 200 µs, single session duration 30 min (25). The stimulation intensity will be individually adjusted based on each participant's sensory threshold. The sensory threshold is defined as the minimum current intensity at which the participant first clearly perceives a tingling sensation on the skin surface (starting from 0.1 mA and increasing slowly). The stimulation intensity in the active taVNS group will be set at a level that is just at or slightly above the sensory threshold (i.e., an intensity that the participant can clearly perceive without pain), typically between 0.5 and 1.5 mA, to ensure a clear, comfortable, and non-painful stimulation sensation. Treatment will be administered once daily, five days per week, for a total of 4 weeks. The sham stimulation group will use the same device, electrode placement, and operational procedures as the active stimulation group. Consistent with previous studies (25), the stimulation current will be set to 0 mA throughout the entire 30 min session. The indicator lights and buzzer sounds of the device will be synchronized with those of the active stimulation group throughout the treatment period.

Both groups will receive standardized conventional rehabilitation training delivered by the same rehabilitation team during the intervention period (weeks 1–4), consisting of 4 weeks, 5 days per week. The training includes: physical therapy (once daily, 40 min): positioning and transfer training (5 min), affected lower limb weight-bearing training (10 min), sit-to-stand transfer training (10 min), balance training (10 min), and walking training (5 min); and occupational therapy (once daily, 30 min): activities of daily living training (e.g., dressing, eating, grooming) and upper limb functional training. All training will be supervised one-on-one by uniformly trained therapists. Training intensity will be assessed every 5 days according to the patient's Brunnstrom stage and modified Barthel Index score, following an individualized progression principle. Training performance will be recorded in a standardized training log and reviewed weekly by the study coordinator. Adjustments to the duration of individual modules based on the patient's functional characteristics are permitted, provided the total duration remains unchanged; however, adding or removing training modules is not allowed. During the follow-up period (weeks 5–8), participants will continue to receive the same conventional rehabilitation training as in the intervention period (with unchanged content, frequency, and supervision), but will no longer receive taVNS or sham stimulation. Training performance will continue to be recorded and reviewed.

### Cardiopulmonary exercise testing

CPET will be performed at the Cardiopulmonary Exercise Laboratory of Shaanxi Rehabilitation Hospital at T0, T1, and T2. All tests will be conducted during the same time window between 9:00 a.m. and 12:00 p.m. A short (5–10 min) low-intensity familiarization session will be conducted 24–48 h before the formal baseline CPET to reduce learning effects. Participants will undergo a symptom-limited CPET on an electromagnetically braked cycle ergometer. For each participant, test conditions (ramp rate, seat position, foot fixation, and equipment configuration) will be kept identical at T0, T1, and T2, and verified by the same technician following a standardized protocol. A linear incremental ramp protocol will be used, with an increment rate of 5–10 W/min and a target test duration of 8–12 min. The specific rate will be selected based on baseline 6 min walk test performance: 5 W/min for ≤300 m, and 8–10 W/min for >300 m. For hemiparetic participants, the affected foot will be secured to the pedal using a fixation strap, and the seat position will be adjusted to approximately 5–10° of hip and knee flexion. If the participant cannot maintain 60 rpm, the cadence may be reduced to 50 rpm, with the actual cadence recorded. Asymmetric pedaling will be addressed through verbal encouragement for bilateral coordination. CPET operators will remain blinded to group allocation and time point. Verbal encouragement will be standardized using phrases such as “Please continue, keep up the rhythm” and “Two more minutes, well done,” with the number of encouragements recorded to ensure consistency across time points. Gas exchange parameters (VO_2_, VCO_2_) will be continuously measured using the breath-by-breath method, with simultaneous 12-lead electrocardiogram and blood pressure monitoring (every 2 min).

The safety termination criteria are: ischemic ECG changes, severe arrhythmias, or abnormal blood pressure (systolic ≥220 mmHg or diastolic ≥110 mmHg). The effort/quality reference criteria (which do not trigger immediate termination) include: respiratory exchange ratio (RER) ≥1.10, heart rate ≥85% of predicted maximum, or appearance of a VO_2_ plateau. The predicted maximum heart rate will be calculated using the formula: HRmax = 220 − age. For patients taking beta-blockers, or those with atrial fibrillation, or post-stroke autonomic dysfunction, the 85% predicted heart rate criterion may not be applicable; in such cases, effort adequacy will be assessed based on a combination of VO_2_ plateau, RER, and the reason for termination, rather than relying on a single heart-rate criterion. Inability to maintain pedaling rate is also a termination indicator. After test termination, the reason for termination will be recorded according to the following classification: cardiopulmonary limitation, neurological or motor limitation, musculoskeletal limitation, safety-related termination, or technical failure. The primary outcome is VO_2_peak, calculated as the average value over the final 30 s before termination. Gas and flow sensors will be calibrated before each test. Data will be acquired and stored using the dedicated SpiroPerfect cardiopulmonary exercise testing software.

VO_2_peak will be reported both as an absolute value (L/min) and relative to body weight (mL/kg/min). For tests terminated prematurely due to symptoms, the average VO_2_ over the final 30 s before termination will be taken as VO_2_peak, and the reason for termination will be recorded. Invalid tests due to non-physiological reasons will be repeated within 48 h. The proportion of participants meeting the criteria for adequate effort, peak work rate, exercise duration, and the reason for termination will be reported to comprehensively assess test quality. The ventilatory threshold will be analyzed as an exploratory outcome when data permit. Adequate effort will be determined based on a combination of VO_2_ plateau, respiratory exchange ratio, heart rate response (≥85% predicted), exercise duration, and the reason for termination, to avoid misclassifying effort as insufficient based on a single criterion (e.g., RER < 1.05). The primary analysis will follow the intention-to-treat principle, with missing data handled using multiple imputation. In the per-protocol analysis, participants who fail to achieve maximal effort (respiratory exchange ratio <1.05 or heart rate <85% of predicted) will be excluded as a sensitivity analysis.

### Heart rate variability acquisition and analysis

As an exploratory outcome, resting-state electrocardiograms (ECGs) will be recorded before cardiopulmonary exercise testing at T0, T1, and T2. Participants will be placed in a supine position with eyes closed and breathing spontaneously, following a 10 min rest period, between 9:00 and 11:00 a.m., with no caffeine, tea, nicotine, or alcohol intake, and at least 2 h fasting. Using a Healink ECG device (Beijing Hailanxin Medical Technology Co., Ltd., China) with a standard V5 lead (positive electrode placed on the left anterior axillary line at the fifth intercostal space, negative electrode on the right midclavicular line at the first intercostal space), a 5 min ECG will be recorded (sampling rate 250 Hz). Artifacts in the RR interval series will be identified using a custom MATLAB program (RR < 300 ms or >1,500 ms, or a difference >50% from the adjacent RR interval), combined with visual inspection by human reviewers. Ectopic beats (premature atrial or ventricular contractions) will be identified by morphology, and their RR intervals will be replaced by interpolation of adjacent normal RR intervals. If the ectopic beat rate exceeds 5%, the corresponding data segment will be excluded from the primary analysis. Atrial fibrillation not identified at baseline and other rhythms unsuitable for conventional HRV analysis will be flagged as non-analyzable, excluded from the primary HRV analysis, and the proportion of excluded recordings will be reported separately (paced rhythms are already excluded by the exclusion criteria). Quality checks will be performed by two independent research assistants, with arbitration in case of disagreement. Time-domain (SDNN) and frequency-domain (HF, 0.15–0.40 Hz, autoregressive model) indices will be calculated using Kubios HRV Standard software (26). Frequency-domain indices will be natural-log transformed (ln-HF) before statistical analysis, while raw values will be reported as median (interquartile range). As an exploratory outcome, HRV will not be adjusted for multiple comparisons and will be used only for mechanistic interpretation.

HRV analysis will be performed by an independent researcher who is blinded to both group allocation (active vs. sham) and time point (T0, T1, T2). All ECG recordings will be anonymized by removing participant IDs, group information, and time point identifiers before batch analysis, so that the analyst will only see anonymous codes.

### Adverse event monitoring

Throughout the trial, participants will be instructed to proactively report any adverse events, including but not limited to: local skin irritation, erythema, dizziness, transient palpitations, nausea, headache, and tinnitus. All reported adverse events will be documented in detail in the case report form (including time of onset, duration, severity, management, and outcome). A serious adverse event is defined as an event that results in death, is life-threatening, requires hospitalization or prolongation of hospitalization, or causes significant disability. Severity will be graded using the CTCAE (Common Terminology Criteria for Adverse Events) (grades 1–5). Causality will be assessed by the investigator (related, possibly related, suspect, or unrelated). Serious adverse events will be reported to the ethics committee within 24 h. If a serious adverse event with a causal relationship to the intervention occurs, the participant will discontinue treatment but will continue to receive safety follow-up and outcome assessments. Their available data will still be included in the intention-to-treat analysis. At the same time, the participant will be immediately referred to the appropriate department for proper medical management. Safety monitoring will be conducted by the study coordinator, who will review adverse events monthly. Serious events will be jointly managed by the principal investigator and the responsible clinician. Given that the intervention is a low-risk, non-invasive transcutaneous electrical stimulation, an independent Data and Safety Monitoring Board will not be established.

### Data collection

Demographic characteristics (age, sex, education level), stroke-related medical history (disease duration, stroke type, lesion location), comorbidities, and current medication lists will be collected for all participants and recorded in the case report form. During the 2-week period before baseline and throughout the trial, medications affecting heart rate, heart rate variability, exercise tolerance, or inflammation (including beta-blockers, calcium-channel blockers, antiarrhythmic agents, anticholinergic medications, antidepressants, sedatives, corticosteroids, and other anti-inflammatory agents) will be maintained at stable doses unless medically indicated otherwise. Any medication adjustments will be documented in detail (drug name, dose, reason for adjustment, and date), and will be included as covariates in the final statistical analysis. The actual duration, intensity, completion rate, and content of rehabilitation training will be recorded separately for each group. Physical activity outside the study and additional rehabilitation interventions will be documented through weekly follow-up interviews. In the final report, the number and reasons for all screening failures will be recorded, particularly the proportion of patients excluded due to inability to complete CPET.

### Outcome measures

#### Primary outcome measure

The primary outcome of this study is VO_2_peak, with the body-weight-normalized value (mL/kg/min) as the unique primary endpoint. It will be assessed by cardiopulmonary exercise testing at three time points: baseline (T0, pre-intervention), post-intervention (T1, after completion of the 4-week treatment), and follow-up (T2, 4 weeks after the end of the intervention). The absolute value (L/min) will be reported as a supportive measure. The primary efficacy time point is T1 (end of intervention), with T2 (end of follow-up) as a secondary time point for assessing persistence of the effect.

#### Secondary outcome measures

Secondary outcomes include: (1) systemic inflammatory markers (IL-6, TNF-α); (2) neurological deficit severity assessed by the National Institutes of Health Stroke Scale (NIHSS); and (3) activities of daily living measured by the modified Barthel Index (MBI). All secondary outcomes will be assessed at T0, T1, and T2. The MBI serves both as an operational basis for rehabilitation intensity adjustment and as a secondary outcome measure. To avoid a feedback loop, the adjusted MBI (accounting for rehabilitation dose) will be used as the primary interpretative measure for this secondary outcome, while the raw MBI will be used only for rehabilitation adjustment and will not serve as a direct basis for outcome interpretation. The NIHSS may have a floor effect in the enrolled population; its score changes will be used only as a safety or exploratory reference and will not serve as a primary efficacy measure.

### Inflammatory marker assessment

At T0, T1, and T2, between 8:00 and 9:00 a.m. (before cardiopulmonary exercise testing), fasting venous blood samples (5 mL, EDTA anticoagulant tube) will be collected from participants after an overnight fast of at least 8 h. Within 30 min after collection, the samples will be centrifuged at 4 °C and 3,000 rpm for 15 min to separate plasma, which will then be aliquoted and stored at −80 °C. Concentrations of interleukin-6 (IL-6) and tumor necrosis factor-alpha (TNF-α) will be uniformly measured using enzyme-linked immunosorbent assay (ELISA; R&D Systems).

### Mechanistic exploration

To explore potential associations between taVNS-mediated improvement in cardiorespiratory fitness and changes in autonomic function, resting-state electrocardiograms (5 min recordings) will be obtained at T0, T1, and T2. Time-domain (SDNN) and frequency-domain (HF) indices of heart rate variability will be analyzed as exploratory outcomes. These indices will not be subjected to multiplicity correction and will be used solely for exploratory purposes, not for causal inference.

Inflammatory markers (IL-6, TNF-α) remain secondary outcome measures, but their mechanistic interpretation is limited to exploratory analysis. No mediation model is prespecified, and these results will not be used for causal inference.

### Safety assessment

Any adverse events occurring during the intervention period, including but not limited to local skin irritation, erythema, dizziness, palpitations, nausea, headache, and tinnitus, will be recorded and reported by the research coordinator. Investigators will assess the causal relationship between adverse events and the intervention, as well as their severity. Serious adverse events will be immediately reported to the ethics committee, and appropriate medical measures will be taken according to clinical needs.

### Sample size calculation

The sample size for this study was estimated based on the group-by-time interaction effect for the primary outcome, VO_2_peak. Given the current lack of direct prior data on the effect of taVNS on VO_2_peak in stroke patients, we referred to the standardized effect size (*d* = 0.58) reported in a randomized controlled trial in healthy populations (25). Considering the greater heterogeneity in stroke patients, a conservative moderate effect size assumption was adopted for sample size planning. The sample size calculation was based on the group-by-time interaction from repeated-measures ANOVA, while the primary analysis will use a linear mixed-effects model; the two approaches are consistent in their handling of repeated-measures data, and LMM is more robust to missing data, so the sample size adequacy is unaffected. *A priori* power analysis was performed using the *F* tests—ANOVA: Repeated measures, between–within interaction module in G * Power 3.1 software, with the following parameters: effect size *f* = 0.25, *α* = 0.05, power (1 − *β*) = 0.80, number of groups = 2, number of measurements = 3, correlation among repeated measures = 0.50, and non-sphericity correction *ε* = 0.75. The effect size conversion was based on the formula *f* = *d*/2 (for two-group comparisons), with *d* = 0.58 (healthy populations) converted to *f* = 0.25 (stroke patients). A conservative estimate was adopted considering the greater heterogeneity expected in stroke patients. The assumed correlation among repeated measures (*ρ* = 0.50) was based on the typical range (0.3–0.7) reported in similar stroke rehabilitation studies, taking the median value; the non-sphericity correction (*ε* = 0.75) was set as a conservative value recommended by the G * Power software manual. Under these assumptions, the required total sample size was estimated to be approximately 128 participants, i.e., approximately 64 per group. Accounting for a 20% dropout rate, a final sample size of 160 participants (80 per group) was planned. Given the current lack of direct data on the effect of taVNS on VO_2_peak in stroke patients, this study adopted a conservative estimate based on the effect size from healthy populations; future research may further define the clinically meaningful difference in this population.

### Quality control and assurance

All research personnel will receive unified training before the trial commences. The inter-rater reliability, assessed by the intraclass correlation coefficient for scale assessments, must reach ≥0.85 before personnel can participate in formal evaluations. Data entry will employ a double-entry verification mechanism. The research coordinator will be responsible for ongoing data monitoring and review, conducting monthly data integrity checks. At least three neurology and cardiology specialists will jointly supervise the enrollment assessment and diagnostic process. To protect participant privacy, only the research team members, ethics committee, and relevant regulatory authorities will have access to participants' personal information and medical records.

Adherence monitoring: Adherence to rehabilitation training will be monitored using the same threshold as for taVNS/sham stimulation (≥80% of planned sessions), and will be recorded separately from the intervention adherence.

### Statistical methods

Statistical analyses will be performed using SPSS 26.0 and R 4.1.0, following the intention-to-treat principle. All tests will be two-sided, with a significance level of *α* = 0.05 unless otherwise specified for multiple comparisons. Statisticians will remain blinded to group allocation until data lock. All randomized participants will be retained in the intention-to-treat analysis. The per-protocol analysis will report the proportion and characteristics of participants who do not meet the effort criteria. Additional stratified analyses will be considered: (1) all randomized participants; (2) those meeting adequate-effort criteria; (3) those terminating due to cardiopulmonary limitation; and (4) those terminating due to neurological or motor limitation, to assess the robustness of the results. For the primary outcome, VO_2_peak will be analyzed using a linear mixed-effects model, with group, time, and their interaction as fixed effects and participant as a random effect (random intercept) to account for within-subject correlations; a random slope may be added if model fitting requires. Age, sex, baseline VO_2_peak, stroke type, disease duration, baseline National Institutes of Health Stroke Scale score, and recruitment source (hospital or community) will be included as prespecified covariates. In the model, the time factor (T0, T1, T2) is used to estimate longitudinal trends, while baseline VO_2_peak is included solely as a covariate to adjust for baseline differences; the two serve different functions and do not constitute redundant adjustment. The covariance structure will be selected from unstructured, compound symmetry, or first-order autoregressive models based on the Akaike information criterion or Bayesian information criterion. The primary treatment effect will be assessed based on the baseline-adjusted between-group difference in VO_2_peak at T1, and the effect estimate, 95% confidence interval, and corresponding *p*-value will be reported. For secondary outcomes, all four measures (interleukin-6, tumor necrosis factor-alpha, National Institutes of Health Stroke Scale, and modified Barthel Index) will be analyzed longitudinally using the same linear mixed-effects model as for the primary outcome. For variables with substantial deviation from normal distribution or violations of parametric assumptions, generalized linear mixed models or nonparametric methods will be used as supplements. To control for multiple comparisons, a Bonferroni correction will be applied for the four secondary outcomes, with the significance threshold set at *α* = 0.05/4 = 0.0125. The exploratory outcome (heart rate variability) will not be adjusted for multiple comparisons and will be used only for exploratory interpretation. For missing data, the primary analysis will use the linear mixed-effects model under the missing-at-random assumption, utilizing all available observed values. To assess robustness, multiple imputation by chained equations and per-protocol analysis will be conducted as sensitivity analyses. In addition, the originally prespecified repeated-measures analysis of variance will be performed as a complementary analysis for comparison with the primary results; if the sphericity assumption is violated, the Greenhouse-Geisser correction will be applied, and *post-hoc* comparisons will be performed using the Bonferroni method. The incidence of adverse events will be compared using Fisher's exact test. If the sample size permits, prespecified exploratory subgroup analyses will be conducted for stroke type (ischemic vs. hemorrhagic) and recruitment source (hospital vs. community), using interaction tests to assess potential effect modification, with results interpreted only as exploratory. Due to sample size constraints (80 participants per group), stratification was performed only for age and disease duration. Other heterogeneity factors, including stroke type, baseline NIHSS score, and baseline VO_2_peak, have been incorporated as prespecified covariates into the linear mixed-effects model for adjustment.

### Ethics and dissemination

This study has been approved by the Ethics Review Committee of Shaanxi Rehabilitation Hospital (initial protocol Version 1.0, Approval No.: 2025079, date of approval: August 7, 2025). A protocol amendment (Version 2.0) changing the sample size from 92 to 160 participants has been filed with the Ethics Committee, with the approval number and date unchanged (2025079, August 7, 2025). The trial registration information is as follows: registration number (ChiCTR2500114436), registration date (December 11, 2025), protocol version (Version 2.0), primary outcome (VO_2_peak normalized to body weight, mL/kg/min), primary time point (T1, end of week 4), and final sample size (160 participants, 80 per group). The planned recruitment period is from July 2026 to June 2027. The trial can be accessed using the registration number on the ChiCTR website (https://www.chictr.org.cn). The changes to the sample size and statistical analysis were made before the start of participant enrollment, and no trial data have been examined.

Before enrollment, all participants will receive face-to-face counseling to fully understand the study purpose, procedures, potential risks, and their rights, after which they will voluntarily sign written informed consent forms. Participants may withdraw from the study unconditionally at any time, with the reasons for withdrawal recorded, and their medical rights will not be affected. The results of this study will be submitted for publication in a peer-reviewed journal.

**SPIRIT guidelines:** This protocol has been prepared in accordance with the SPIRIT (Standard Protocol Items: Recommendations for Interventional Trials) guidelines. The completed SPIRIT checklist has been submitted as a Supplementary File.

## Discussion

We hypothesize that taVNS may improve cardiorespiratory fitness in stroke patients through the following potential pathways. Stroke often leads to sympathetic-parasympathetic imbalance, manifested as increased resting heart rate and reduced heart rate variability (27, 28). TaVNS may theoretically enhance vagal efferent activity, thereby contributing to the restoration of autonomic balance, optimizing cardiac chronotropic function, and improving cardiac function (29). The vagal anti-inflammatory pathway is considered a crucial bridge between the nervous and cardiovascular systems (30, 31), and taVNS may inhibit the release of peripheral inflammatory markers through activation of this pathway, thereby improving energy metabolism in skeletal and cardiac muscle (32, 33). In addition, the nucleus tractus solitarius has extensive fiber connections with the medulla, midbrain, and cortical motor areas; repeated taVNS stimulation may indirectly promote motor function recovery through neuroplasticity mechanisms.

Previous studies in healthy populations have suggested that taVNS may increase VO_2_peak and reduce inflammatory responses (25). This protocol attempts to extend these findings to the domain of cardiorespiratory rehabilitation in patients with subacute stroke. It should be noted that VO_2_peak is a surrogate/functional endpoint, not a direct measure of clinical cardiovascular outcomes such as myocardial infarction or cardiovascular death. VO_2_peak only reflects the highest value achieved by the patient during that specific test and does not represent a true physiological cardiopulmonary limit.

Currently, standardized stimulation parameters for taVNS in stroke rehabilitation are lacking. The parameter settings in this study (frequency 25 Hz, pulse width 200 µs, intensity set at or slightly above the sensory threshold) are based on parameter combinations previously validated as effective in earlier research (25), with adaptive adjustments considering the skin sensitivity of stroke patients and the anatomical characteristics of the auricular concha. These parameters were selected to balance neuromodulatory efficacy and patient tolerability.

Reduced cardiorespiratory fitness represents a significant obstacle to functional recovery after stroke and serves as an independent predictor of adverse cardiovascular prognosis (6). Conventional rehabilitation training has limited efficacy in improving cardiorespiratory fitness. If this study suggests the efficacy of taVNS, it may provide a clinically applicable adjunctive treatment that is early-interventional, easy to administer, and associated with high compliance, which theoretically could shorten the rehabilitation course.

### Limitations

This study has several limitations. First, the single-center design and relatively limited sample size may affect the generalizability of the findings. Second, although rigorous blinding procedures have been implemented (participants, clinicians, outcome assessors, and statisticians will remain blinded), the intervention implementers cannot be blinded due to the visibility of the stimulation device, which may introduce potential performance bias. Third, the stimulation parameters have not been fully optimized, and a single parameter combination may not adequately address the heterogeneity of responses across all patients. Fourth, the follow-up period is relatively short (4 weeks post-intervention), precluding assessment of long-term efficacy and preventive effects on hard cardiovascular endpoints after stroke (e.g., cardiovascular death, myocardial infarction, heart failure hospitalization). Fifth, although this study employs a sham stimulation control and incorporates blinding assessment, it remains impossible to completely rule out the possibility that participants may guess their group allocation based on perceived differences in sensation. To monitor blinding effectiveness, participants will be asked about their group guess (active stimulation, sham stimulation, or uncertain) at the end of each week during the treatment period. The proportion of correct guesses will be calculated and analyzed using a binomial test to determine whether it is significantly higher than 50%. If the correct guess rate at any assessment point is significantly higher than 50%, this will be reported as a limitation. It should be noted that this assessment is intended only to monitor blinding effectiveness and cannot substitute for a credible sham stimulation design. Sixth, respiratory rate and tidal volume were not controlled during HRV acquisition, which may affect the accuracy of high-frequency HRV measurements. Both HRV and inflammatory markers are exploratory analytical measures; their results are intended only to explore potential associations and cannot be used for causal inference. In addition, improvement in VO_2_peak is influenced by multiple factors and should not be directly attributed to improvements in autonomic or cardiac mechanisms. Seventh, the study population consists of patients with mild-to-moderate subacute stroke who are able to complete cardiopulmonary exercise testing. Therefore, the results cannot be directly generalized to patients with severe hemiparesis, bedridden patients, patients with substantial cognitive impairment, or those unable to complete CPET. Eighth, this study did not include a specific assessment of lower-limb motor function, and the association between improvements in VO_2_peak and motor recovery remains unclear. Future studies may consider incorporating lower-limb motor function measures, such as the Fugl–Meyer lower extremity scale or the 10 m walk test.

### Future directions

Future research should proceed in the following directions: conducting multicenter, large-sample randomized controlled trials to validate the efficacy of taVNS in patients at different stages and with different stroke types; exploring the effects of different stimulation parameters on the efficacy of taVNS to optimize the treatment regimen; extending follow-up periods and incorporating major adverse cardiovascular events (cardiovascular death, non-fatal myocardial infarction, heart failure hospitalization) as core hard endpoints to verify the improving effect of taVNS on post-stroke cardiovascular prognosis; and simultaneously employing functional magnetic resonance imaging or near-infrared spectroscopy to elucidate the central mechanisms through which taVNS improves cardiorespiratory fitness. Furthermore, exploring more perceptually matched sham stimulation designs to further optimize blinding methodology is also a worthwhile direction for future research.

## Conclusion

This protocol aims to investigate the effects of taVNS on cardiorespiratory fitness in patients with subacute stroke. If the results are positive, they may provide preliminary evidence for taVNS as a post-stroke cardiorespiratory rehabilitation strategy and expand the intervention dimensions of stroke rehabilitation.

## References

[1] Wang Y Lv H He M Wu P Li F Wang Y. Correlation analysis of activity levels and risk factors in patients with stroke: variations in cardiac function according to the Longshi scale. J Multidiscip Healthc. (2024) 17:4757–67. 10.2147/JMDH.S479131 39431061 PMC11490244

[2] Sposato LA Hilz MJ Aspberg S Murthy SB Bahit MC Hsieh C-Y. Post-stroke cardiovascular complications and neurogenic cardiac injury: jACC state-of-the-art review. J Am Coll Cardiol. (2020) 76(23):2768–85. 10.1016/j.jacc.2020.10.009 33272372

[3] Scheitz JF Sposato LA Schulz-Menger J Nolte CH Backs J Endres M. Stroke-heart syndrome: recent advances and challenges. J Am Heart Assoc. (2022) 11(17):e026528. 10.1161/JAHA.122.026528 36056731 PMC9496419

[4] Baricich A Borg MB Battaglia M Facciorusso S Spina S Invernizzi M. High-intensity exercise training impact on cardiorespiratory fitness, gait ability, and balance in stroke survivors: a systematic review and meta-analysis. J Clin Med. (2024) 13(18):5498. 10.3390/jcm13185498 39336984 PMC11432212

[5] Saunders DH Greig CA Mead GE. Physical activity and exercise after stroke: review of multiple meaningful benefits. Stroke. (2014) 45(12):3742–7. 10.1161/STROKEAHA.114.004311 25370588

[6] American Thoracic Society, American College of Chest Physicians. ATS/ACCP statement on cardiopulmonary exercise testing. Am J Respir Crit Care Med. (2003) 167(2):211–77. 10.1164/rccm.167.2.211 12524257

[7] Zhao Y Sun H Qie R Han M Zhang M Shi X. Association between cardiorespiratory fitness and risk of all-cause and cause-specific mortality. Eur J Clin Invest. (2022) 52(7):e13770. 10.1111/eci.13770 35294786

[8] Ross R Blair SN Arena R Church TS Després J-P Franklin BA. Importance of assessing cardiorespiratory fitness in clinical practice: a case for fitness as a clinical vital sign: a scientific statement from the American Heart Association. Circulation. (2016) 134(24):e653–99. 10.1161/CIR.0000000000000461 27881567

[9] Luo L Meng H Wang Z Zhu S Yuan S Wang Y. Effect of high-intensity exercise on cardiorespiratory fitness in stroke survivors: a systematic review and meta-analysis. Ann Phys Rehabil Med. (2020) 63(1):59–68. 10.1016/j.rehab.2019.07.006 31465865

[10] Rebeiz T Sabirov T White TG Ledoux D Kim J- Kerner D. Noninvasive vagus nerve stimulation in spontaneous subarachnoid hemorrhage (VANQUISH): a randomized safety and feasibility study. Brain Stimul. (2024) 17(3):543–9. 10.1016/j.brs.2024.04.004 38641171

[11] Zhang J Zhou F Roberts N Yuan Q Wang M Wu G. Invasive or non-invasive vagus nerve stimulation modulation of brain function and remodeling after stroke: a review. Int J Surg. (2025) 111(2):2148–61. 10.1097/JS9.0000000000002173 39715153

[12] Ryvlin P Rheims S Hirsch LJ Sokolov A Jehi L. Neuromodulation in epilepsy: state-of-the-art approved therapies. Lancet Neurol. (2021) 20(12):1038–47. 10.1016/S1474-4422(21)00300-8 34710360

[13] Conway CR Rush AJ Aaronson ST Bunker MT Gordon C George MS. Durability of the benefit of vagus nerve stimulation in markedly treatment-resistant major depression: a RECOVER trial report. Int J Neuropsychopharmacol. (2026) 29(1):yaf080. 10.1093/ijnp/pyaf080 PMC12799215 41529263

[14] Xiong X Tao M Zhao W Tan W Jiang Y Sun Z. Transcutaneous auricular vagus nerve stimulation for postpartum contraction pain during elective cesarean delivery: a randomized clinical trial. JAMA Netw Open. (2025) 8(8):e2529127. 10.1001/jamanetworkopen.2025.29127 40880089 PMC12397895

[15] Baker MC Kavanagh S Cohen S Matsumoto AK Dikranian A Tesser J. A randomized, double-blind, sham-controlled, clinical trial of auricular vagus nerve stimulation for the treatment of active rheumatoid arthritis. Arthritis Rheumatol. (2023) 75(12):2107–15. 10.1002/art.42637 37390360

[16] Dawson J Liu CY Francisco GE Cramer SC Wolf SL Dixit A. Vagus nerve stimulation paired with rehabilitation for upper limb motor function after ischaemic stroke (VNS-REHAB): a randomised, blinded, pivotal, device trial. Lancet. (2021) 397(10284):1545–53. 10.1016/S0140-6736(21)00475-X 33894832 PMC8862193

[17] Stavrakis S Stoner JA Humphrey MB Morris L Filiberti A Reynolds JC. TREAT AF (transcutaneous electrical vagus nerve stimulation to suppress atrial fibrillation): a randomized clinical trial. JACC Clin Electrophysiol. (2020) 6(3):282–91. 10.1016/j.jacep.2019.11.008 32192678 PMC7100921

[18] Elkholey K Niewiadomska M Morris L Whyte S Houser J Humphrey MB. Transcutaneous vagus nerve stimulation ameliorates the phenotype of heart failure with preserved ejection fraction through its anti-inflammatory effects. Circ Heart Fail. (2022) 15(8):e009288. 10.1161/CIRCHEARTFAILURE.122.009288 35862007 PMC9388556

[19] Stavrakis S Elkholey K Morris L Niewiadomska M Asad ZUA Humphrey MB. Neuromodulation of inflammation to treat heart failure with preserved ejection fraction: a pilot randomized clinical trial. J Am Heart Assoc. (2022) 11(3):e023582. 10.1161/JAHA.121.023582 35023349 PMC9238491

[20] Guazzi M Dickstein K Vicenzi M Arena R. Six-minute walk test and cardiopulmonary exercise testing in patients with chronic heart failure: a comparative analysis on clinical and prognostic insights. Circ Heart Fail. (2009) 2(6):549–55. 10.1161/CIRCHEARTFAILURE.109.881326 19919979

[21] Fletcher GF Ades PA Kligfield P Arena R Balady GJ Bittner VA. Exercise standards for testing and training: a scientific statement from the American Heart Association. Circulation. (2013) 128(8):873–934. 10.1161/CIR.0b013e31829b5b44 23877260

[22] Feng W Hendry RM Adams RJ. Risk of recurrent stroke, myocardial infarction, or death in hospitalized stroke patients. Neurology. (2010) 74(7):588–93. 10.1212/WNL.0b013e3181cff776 20157161

[23] Ruigrok TJH Mantel SA Orlandini L De Knegt C Vincent AJPE Spoor JKH. Sympathetic components in left and right human cervical vagus nerve: implications for vagus nerve stimulation. Front Neuroanat. (2023) 17:1205660. 10.3389/fnana.2023.1205660 37492698 PMC10364449

[24] Zanello M Voges B Chelvarajah R Sen A Petelin Gadže Ž Penchet G. Right-sided vagus nerve stimulation: worldwide collection and perspectives. Ann Clin Transl Neurol. (2025) 12(3):565–76. 10.1002/acn3.52312 39901698 PMC11920732

[25] Ackland GL Patel ABU Miller S Gutierrez del Arroyo A Thirugnanasambanthar J Ravindran JI. Non-invasive vagus nerve stimulation and exercise capacity in healthy volunteers: a randomized trial. Eur Heart J. (2025) 46(17):1634–44. 10.1093/eurheartj/ehaf037 39969124 PMC7617618

[26] Lin P-Y Tsai C-T Hsu CF Lee Y-H Huang H-P Huang C-C. The autonomic imbalance of myocardial ischemia during exercise stress testing: insight from short-term heart rate variability analysis. Int J Environ Res Public Health. (2022) 19(22):15096. 10.3390/ijerph192215096 36429812 PMC9690482

[27] Sandu OE Bogdan C Apostol A Simu MA Moga V-D Pecingina R-M. Heart rate variability dynamics as predictors of functional recovery and mortality after acute ischemic stroke. Biomedicines. (2025) 13(9):2217. 10.3390/biomedicines13092217 41007779 PMC12467282

[28] Lago S De Beukelaar TT Casetta I Arcara G Mantini D. Heart rate variability and autonomic dysfunction after stroke: prognostic markers for recovery. Biomedicines. (2025) 13(7):1659. 10.3390/biomedicines13071659 40722730 PMC12292726

[29] De Ferrari GM Crijns HJGM Borggrefe M Milasinovic G Smid J Zabel M. Chronic vagus nerve stimulation: a new and promising therapeutic approach for chronic heart failure. Eur Heart J. (2011) 32(7):847–55. 10.1093/eurheartj/ehq391 21030409

[30] Huo J-Y Hou C Jia F Xue C Li X-L Yang L. Transcutaneous auricular vagus nerve stimulation ameliorates heart failure with preserved ejection fraction through regulating macrophage polarization mediated by alpha7nAChR. Cardiovasc Drugs Ther. (2026) 40(1):127–40. 10.1007/s10557-025-07695-0 40193005

[31] Bazoukis G Stavrakis S Armoundas AA. Vagus nerve stimulation and inflammation in cardiovascular disease: a state-of-the-art review. J Am Heart Assoc. (2023) 12(19):e030539. 10.1161/JAHA.123.030539 37721168 PMC10727239

[32] Xin Y Zhang Y Deng S Hu X. Vagus nerve stimulation attenuates acute skeletal muscle injury induced by hepatic ischemia/reperfusion injury in rats. Front Pharmacol. (2022) 12:756997. 10.3389/fphar.2021.756997 35046803 PMC8762262

[33] Luo B Wu Y Liu S- Li X- Zhu H- Zhang L. Vagus nerve stimulation optimized cardiomyocyte phenotype, sarcomere organization and energy metabolism in infarcted heart through FoxO3A-VEGF signaling. Cell Death Dis. (2020) 11(11):971. 10.1038/s41419-020-03142-0 33184264 PMC7665220

