# Supplementary material for: Effects of transcutaneous auricular vagus nerve stimulation on cardiorespiratory fitness in patients with subacute stroke: a study protocol for a randomized controlled trial
Source: Front Cardiovasc Med. 2026 Aug 11;13:1835286. doi: 10.3389/fcvm.2026.1835286 (PMC13503236; doi:10.3389/fcvm.2026.1835286)
Supplement: Supplementary file 1 [file Table1.doc]

**SPIRIT Checklist**

**Study protocol title:** Effects of Transcutaneous Auricular Vagus Nerve Stimulation on Cardiorespiratory Fitness in Patients with Subacute Stroke: A Study Protocol for a Randomized Controlled Trial

| Item | Section | Content | Location in manuscript |
| --- | --- | --- | --- |
| **I. Administrative information** |  |  |  |
| 1 | Title | Effects of Transcutaneous Auricular Vagus Nerve Stimulation on Cardiorespiratory Fitness in Patients with Subacute Stroke: A Study Protocol for a Randomized Controlled Trial | Title page |
| 2 | Trial registration | ChiCTR2500114436; Registration date: December 11, 2025 | Methods/Study Design |
| 3 | Protocol version | Protocol amendment Version 2.0 (sample size changed from 92 to 160 participants) | Ethics and Dissemination |
| 4 | Funding source | Shaanxi Provincial Rehabilitation Hospital | End of manuscript |
| 5 | Conflict of interest | The authors declare no competing interests | End of manuscript |
| **II. Introduction** |  |  |  |
| 6a | Background and rationale | Pathophysiology of reduced cardiorespiratory fitness after stroke, potential role of taVNS, gaps in current evidence | Introduction |
| 6b | Study objectives | To evaluate the efficacy of 4-week taVNS combined with conventional rehabilitation on cardiorespiratory fitness (VO₂peak) in patients with subacute stroke | End of Introduction |
| **III. Methods** |  |  |  |
| 7 | Study design | Randomized, sham-controlled, parallel-group superiority trial | Methods/Study Design |
| 8a | Inclusion criteria | Age 40-75 years; first-ever stroke, disease duration 1-6 months; lower limb hemiparesis Brunnstrom stage ≥ II; affected lower limb muscle strength ≥ grade 3; ability to perform seated pedaling motion; informed consent | Methods/Inclusion Criteria |
| 8b | Exclusion criteria | Severe cardiac, hepatic, or renal dysfunction; uncontrolled hypertension; severe arrhythmia; ear skin lesions/infection/deformity; affected lower limb muscle strength ≤ grade 2; implanted electronic devices; participation in other clinical trials | Methods/Exclusion Criteria |
| 9 | Discontinuation/withdrawal criteria | Serious adverse event (discontinue treatment, included in ITT); voluntary withdrawal of consent (withdraw from study, cease data collection); taVNS/sham adherence <80% (discontinue treatment, continue follow-up); receipt of other interventions (discontinue treatment, included in ITT); loss to follow-up (included in ITT, multiple imputation) | Methods/Discontinuation and Withdrawal Criteria |
| 10 | Interventions | Experimental group: active taVNS (25 Hz, 200 μs, at sensory threshold level, 30 min/session, 5 sessions/week for 4 weeks); Control group: 0 mA sham stimulation; both groups receive standardized conventional rehabilitation (weeks 1-8) | Methods/Intervention |
| 11a | Primary outcome | VO₂peak (peak oxygen uptake), assessed at T0, T1, and T2 | Outcomes/Primary Outcome Measure |
| 11b | Secondary outcomes | Inflammatory markers (IL-6, TNF-α), NIHSS, modified Barthel Index, assessed at T0, T1, and T2 | Outcomes/Secondary Outcome Measures |
| 11c | Exploratory outcome | Heart rate variability (SDNN, HF), for mechanistic interpretation | Outcomes/Mechanistic Exploration |
| 12 | Randomization and allocation | Stratified block randomization (age ≤60/>60 years, disease duration ≤3/>3 months); 1:1 allocation to experimental group (n=80) and control group (n=80) | Methods/Randomization and Allocation |
| 13 | Blinding | Partial blinding: participants, clinicians, outcome assessors, and statisticians remain blinded; intervention providers cannot be blinded due to visibility of stimulation site | Methods/Allocation and Blinding |
| 14a | Data collection and follow-up time points | T0 (baseline), T1 (end of week 4), T2 (end of week 8) | Table 1, Table 2, Methods section |
| 14b | Data collection content | Demographic characteristics, medical history, concomitant medications, adverse events, outcome measures | Methods/Data Collection, Outcomes section |
| 15 | Sample size estimation | Effect size f = 0.25, α = 0.05, power (1-β) = 0.80, number of repeated measurements = 3, correlation ρ = 0.5, non-sphericity correction ε = 0.75; 64 participants per group, 20% dropout, final 80 per group, total 160 | Sample Size Calculation |
| 16a | Statistical methods (primary) | Linear mixed-effects model (fixed effects: group, time, interaction; random effect: participant intercept), with covariates | Statistical Methods |
| 16b | Statistical methods (secondary/exploratory) | Secondary outcomes analyzed using same mixed model; four secondary outcomes (IL-6, TNF-α, NIHSS, MBI) with Bonferroni correction, α = 0.0125; exploratory outcome (HRV) not adjusted for multiple comparisons | Statistical Methods |
| 16c | Missing data handling | Assumed missing at random, multiple imputation by chained equations, with per-protocol analysis as sensitivity analysis | Statistical Methods |
| 16d | Subgroup analyses | Stroke type (ischemic vs. hemorrhagic), recruitment source (hospital vs. community), if sample size permits | Statistical Methods |
| 16e | Sensitivity analysis | Repeated-measures analysis of variance, per-protocol analysis | Statistical Methods |
| **IV. Ethics and dissemination** |  |  |  |
| 17 | Ethics approval | Ethics Review Committee of Shaanxi Rehabilitation Hospital (Approval No.: 2025079) | Ethics and Dissemination |
| 18 | Informed consent | Face-to-face explanation of the study, voluntary signing of written informed consent | Ethics and Dissemination |
| 19 | Data monitoring | Study coordinator performs monthly data integrity checks | Quality Control and Assurance |
| 20 | Adverse event management | Adverse events recorded and reported; serious adverse events immediately reported to ethics committee, participant discontinues treatment and is referred for appropriate care | Adverse Event Monitoring |
| 21 | Publication policy | Results will be submitted for publication in a peer-reviewed journal, regardless of outcome | Ethics and Dissemination |
| 22 | Data sharing | Not applicable (or contact corresponding author) | Not applicable |
| 23 | Protocol amendments | Protocol amendment Version 2.0 documented; ChiCTR will be updated before enrollment | Sample Size Calculation/Ethics and Dissemination |
| 24 | Informed consent materials | Participant recruitment materials (poster, recruitment flyer) provided | Appendix |
| **V. Other** |  |  |  |
| 25 | Data collection tools | Case report form (CRF), standardized training log, cardiopulmonary exercise testing software, etc. | Methods/Data Collection, Quality Control |
| 26 | Biological sample management | Blood collection, centrifugation, aliquoting, storage at -80°C; uniform ELISA testing | Outcomes/Inflammatory Marker Assessment |
| 27 | Quality control | Unified researcher training, ICC ≥ 0.85, double data entry, regular data review; adherence to rehabilitation training monitored using same threshold as taVNS/sham (≥80% of planned sessions), recorded separately from intervention adherence | Quality Control and Assurance |
| 28 | Patient and public involvement | Not applicable | Not applicable |
| 29 | Protocol change record | Sample size changed from 92 to 160 participants (Protocol Amendment Version 2.0) | Sample Size Calculation |
| 30 | Interim analysis | Not applicable | Not applicable |
| 31 | Blinding of outcome assessment | HRV analysts blinded to both group allocation and time point | Heart Rate Variability Acquisition and Analysis |
| 32 | Data confidentiality | Only research team members, ethics committee, and relevant regulatory authorities have access to participants’ personal information | Quality Control and Assurance |
| 33 | Sample size assumptions | Effect size f = 0.25, ρ = 0.5, ε = 0.75, dropout rate 20% | Sample Size Calculation |
| 34 | Informed consent special circumstances | Not applicable | Not applicable |

**Declaration:** This protocol has been prepared in accordance with the SPIRIT 2025 guidelines. The completed SPIRIT checklist has been submitted as a supplementary file.
